# Efficacy and safety evaluation of Allisartan Isoproxil in patients with hypertension: a meta-analysis

**DOI:** 10.3389/fcvm.2024.1355014

**Published:** 2024-06-06

**Authors:** Fengfeng Zhao, Yihua Liu, Liang Chen

**Affiliations:** ^1^ School of Clinical Medicine, Shandong Second Medical University, Weifang, Shandong, China; ^2^ Department of Adult Internal Medicine, Qingdao Women’s and Children’s Hospital, Qingdao, Shandong, China

**Keywords:** Allisartan Isoproxil, hypertension, meta-analysis, systematic review, randomized controlled trial

## Abstract

**Objective:**

This study aimed to evaluate the effectiveness and safety of Allisartan Isoproxil in the management of hypertension.

**Methods:**

A comprehensive search was conducted across both English and Chinese databases, including the Cochrane Library, Embase, PubMed, Web of Science, Chinese Journal Full Text Database (CNKI), Wanfang Digital Periodical Full Text Database, and VIP Chinese Periodical Database (VIP), up to March 24, 2024. Randomized controlled trials (RCTs) investigating alisartan axetil for hypertension management were selected. Literature quality was assessed, and data were extracted for meta-analysis using Stata 15.1 software. The quality of evidence for outcome indicators was evaluated using the GRADE system level.

**Results:**

Six RCTs involving 767 participants were included. Meta-analysis revealed that, compared to placebo, the Allisartan Isoproxil group exhibited a significant reduction in systolic blood pressure (SBP) [WMD = −8.08, 95% CI (−11.81, 4.10), *p *= 0.000] and brachial-ankle pulse wave velocity (baPWV) [SMD = −0.69, 95% CI (−1.17, 0.20), *p* = 0.006]. However, the reduction in diastolic blood pressure (DBP) was not statistically significant [WMD = −5.48, 95% CI (−11.07, 0.10), *p* = 0.054]. Additionally, compared to calcium channel blockers (CCB) and angiotensin II receptor blockers (ARB), Allisartan Isoproxil did not significantly affect SBP [WMD = 0.20, 95% CI (−3.71, 4.10), *p* = 0.921] or DBP [WMD = 0.16, 95% CI (−2.11, 2.43), *p* = 0.891]. Allisartan Isoproxil demonstrated superior effects in increasing nitric oxide (NO) levels and decreasing endothelin (ET) levels compared to control groups [WMD = 9.56, 95% CI (6.42, 12.71), *p *= 0.000], [WMD = −7.42, 95% CI (−11.13, −3.71), *p* = 0.000], and showed a higher effective control rate of blood pressure [RR = 1.26, 95% CI (1.13, 1.41), *p* = 0.000]. Subgroup analysis did not reveal significant differences. Regarding safety, there were no statistically significant differences in adverse events between the Allisartan Isoproxil group and the control groups [RR = 0.99, 95% CI (0.74, 1.32), *p* = 0.928], and no fatal adverse events were reported.

**Conclusion:**

Allisartan Isoproxil is effective in reducing SBP and baPWV, increasing NO, decreasing ET, and achieving a higher control rate of blood pressure in patients with essential hypertension. These benefits are achieved with minimal adverse reactions.

**Systematic Review Registration:**

https://www.crd.york.ac.uk/prospero/display_record.php?ID=CRD42023467869, identifier PROSPERO CRD42023467869.

## 1 Introduction

Recent findings from the Global Cardiovascular Risk Consortium underscore the pivotal role of elevated blood pressure as the leading risk factor contributing to cardiovascular disease globally (1, 2). Over the past four decades, the prevalence of hypertension has surged by 90%, primarily due to aging demographics and unhealthy lifestyles (3, 4). However, the levels of recognition, management, and control of hypertension remain notably low, especially in low-income and middle-income countries (5–7). A prospective study examining the correlation between blood pressure and cardiovascular outcomes revealed a concerning association: each additional 20 mmHg in systolic blood pressure (SBP) or 10 mmHg in diastolic blood pressure (DBP) doubles the likelihood of cardiovascular events, surpassing the 115/75 mmHg threshold (8, 9). Antihypertensive treatment has consistently demonstrated a significant impact on reducing mortality and cardiovascular disease risks (10–12).

As a chronic condition lacking a cure, hypertension necessitates effective, long-term, and comprehensive management strategies, emphasizing the importance of strengthening patients' self-care capabilities (13–15). Current hypertension management predominantly relies on a combination of antihypertensive medications and lifestyle modifications (16). Nevertheless, clinical challenges persist, including patient intolerance, adverse drug reactions, and insufficient control of blood pressure (17–20). The Renin-angiotensin-aldosterone system (RAAS) plays a crucial role in the pathogenesis of hypertension, prompting the latest guidelines to recommend angiotensin II receptor blockers (ARBs) as first-line therapy (21, 22). By inhibiting Ang II binding to the angiotensin II type 1 receptor (AT1R), ARBs effectively mitigate vascular resistance, reduce blood pressure, and impede RAAS activation, thereby protecting target organs and preventing cardiovascular endpoint events (23, 24).

Allisartan Isoproxil, a novel class 1.1 oral antihypertensive drug, represents a promising candidate in hypertension therapy. Following hydrolysis by esterase in the gastrointestinal tract, Allisartan Isoproxil is promptly converted to the active metabolite EXP3174 upon absorption. EXP3174 selectively binds with AT1R, effectively blocking angiotensin II-induced vascular tension response and thereby exerting its antihypertensive effect (25). Preclinical studies have demonstrated its ability to lower blood pressure, mitigate inflammation and oxidative stress, and improve cardiac remodeling and cardiac dysfunction with minimal toxicity (26). Several clinical studies have demonstrated the favorable antihypertensive impact of Allisartan Isoproxil on mild to moderate essential hypertension, highlighting its safety and effectiveness in blood pressure reduction and organ protection (27, 28). Nonetheless, despite these promising outcomes, the existing evidence base remains inadequately substantiated. Therefore, this study aims to conduct a comprehensive systematic review to evaluate the antihypertensive efficacy and safety of Allisartan Isoproxil in hypertension therapy. By synthesizing data from recent clinical studies and various prognostic markers, this review aims to establish a robust foundation for informing clinical practice and guiding future research in the field of hypertension therapy.

## 2 Methods

This systematic review strictly adhered to the latest PRISMA 2020 checklist guidelines, ensuring transparent reporting of its methodology (29). The protocol for this review was registered in the International Prospective Register of Systematic Reviews (PROSPERO) database under the identifier CRD42023467869.

### 2.1 Literature inclusion criteria

The following were the inclusion criteria: Firstly, individuals diagnosed with hypertension (30), regardless of age, gender, duration of illness, race, or nationality, with a minimum age requirement of 18 years. Secondly, comparative analysis of the efficacy and safety of Allisartan Isoproxil (at any dose) vs. a control group (placebo or no Allisartan Isoproxil) in hypertensive patients. Thirdly, primary efficacy measures included SBP, DBP, and drug-related adverse events (AEs) observed during treatment. Secondary outcomes included assessments of vascular endothelial function, endothelial dysfunction, vascular inflammation, and increased arterial stiffness during treatment. Fourthly, only randomized controlled trials (RCTs) were included.

### 2.2 Literature exclusion criteria

The review excluded studies based on the following criteria: Firstly, non-RCT study types. Secondly, duplicate publication of the same experimental data. Thirdly, incomplete data from meeting documents and research.

### 2.3 Literature screening methods and data sources

We conducted comprehensive searches across several databases, including PubMed, EMbase, the Cochrane Library, and Web of Science for English-language sources, as well as the Chinese Journal Full Text Database (CNKI), Wanfang Digital Periodical Full Text Database, and VIP Chinese Periodical Database (VIP) for Chinese-language sources. The search period covered from the inception of these databases to March 24, 2024. To identify potential articles, we used a combination of Medical Subject Headings (MeSH) and non-MeSH terms. Specific search terms included: (“Hypertension” OR “Hypertensions” OR “Blood Pressure, High” OR “Blood Pressures, High” OR “High Blood Pressure” OR “High Blood Pressures”) AND (“Allisartan Isoproxil” OR “SALS-3 compound”). Detailed search strategies for each database are provided in Supplementary Table S1. Additionally, we manually reviewed the references cited in previously published systematic reviews on related topics to ensure comprehensive coverage of relevant studies. In cases where original data were missing, we contacted the authors for data retrieval. If we did not receive a timely response, the relevant data were excluded.

### 2.4 Data extraction and outcome measures

Two experienced researchers independently conducted a meticulous literature review following predefined inclusion and exclusion criteria. Data collection and risk of bias assessment were performed, with any discrepancies resolved through discussion or involvement of a third investigator. The initial screening involved evaluating titles and abstracts to exclude irrelevant studies, followed by a full-text review to assess research data integrity and accessibility. Data extraction included author information, subject demographics, sample size, intervention methods, treatment duration, outcome measures, and related details, alongside components for evaluating document quality and precise outcome indicator values.

### 2.5 Assessment of risk of bias

The risk of bias was evaluated according to the latest guidelines outlined in the Cochrane Handbook for Systematic Reviews of Interventions. This assessment tool encompassed five primary components: bias stemming from randomization, bias arising from deviations in the intervention, incomplete outcome data, measurement of outcomes, and selective reporting of outcomes. Each study was categorized as having a “low risk of bias,” “some concerns,” or “high risk of bias.” The evaluation was independently conducted by two researchers, with any disagreements resolved through discussion or consultation with a third investigator.

### 2.6 Outcome measures

Primary outcomes included SBP and DBP, while secondary outcomes encompassed brachial-ankle pulse wave velocity (baPWV), nitric oxide (NO), endothelin (ET), and effective blood pressure control rate. Effective blood pressure control was defined as SBP/DBP values below 140 mmHg/90 mmHg or a reduction of at least 20 mmHg in SBP and/or 10 mmHg in DBP (31, 32). Safety indicators encompassed the assessment of AE related to drug treatment during the study.

### 2.7 Data synthesis and statistical analysis

Statistical analysis was performed using Stata 15.1 software. Continuous variables were analyzed using either the weighted mean difference (WMD) or the standardized mean difference (SMD). Binary categorical variables were analyzed using relative risk (RR) as the statistical measure. Each effect size was accompanied by its 95% confidence interval (95% CI). Heterogeneity among the included studies was evaluated using the *I*^2^ statistic. For studies with low statistical heterogeneity (*I*^2^ ≤ 50%, *p* > 0.10), a fixed-effect model was used for the meta-analysis. In cases of significant heterogeneity, further investigation was conducted, and a random-effects model was employed to account for clinical heterogeneity. The significance level for meta-analysis tests was set at *α* = 0.05. Subgroup analyses or sensitivity analyses were performed to address clinical heterogeneity, and Egger's test was used to determine publication bias.

The quality of evidence for each outcome was evaluated using GRADEpro GDT software (33). RCT were initially considered high quality. The quality of evidence was then adjusted based on factors that could downgrade or upgrade the rating. Downgrade factors included risk of bias, inconsistency, indirectness, imprecision, and publication bias. Upgrade factors included a large effect size, evidence of a dose-response gradient, and accounting for plausible confounding factors. Evidence quality was categorized into four levels: high, moderate, low, and very low. A summary table was generated to provide an overview of the findings and their reliability.

## 3 Results

### 3.1 Literature screening process and results

**Figure 1:**
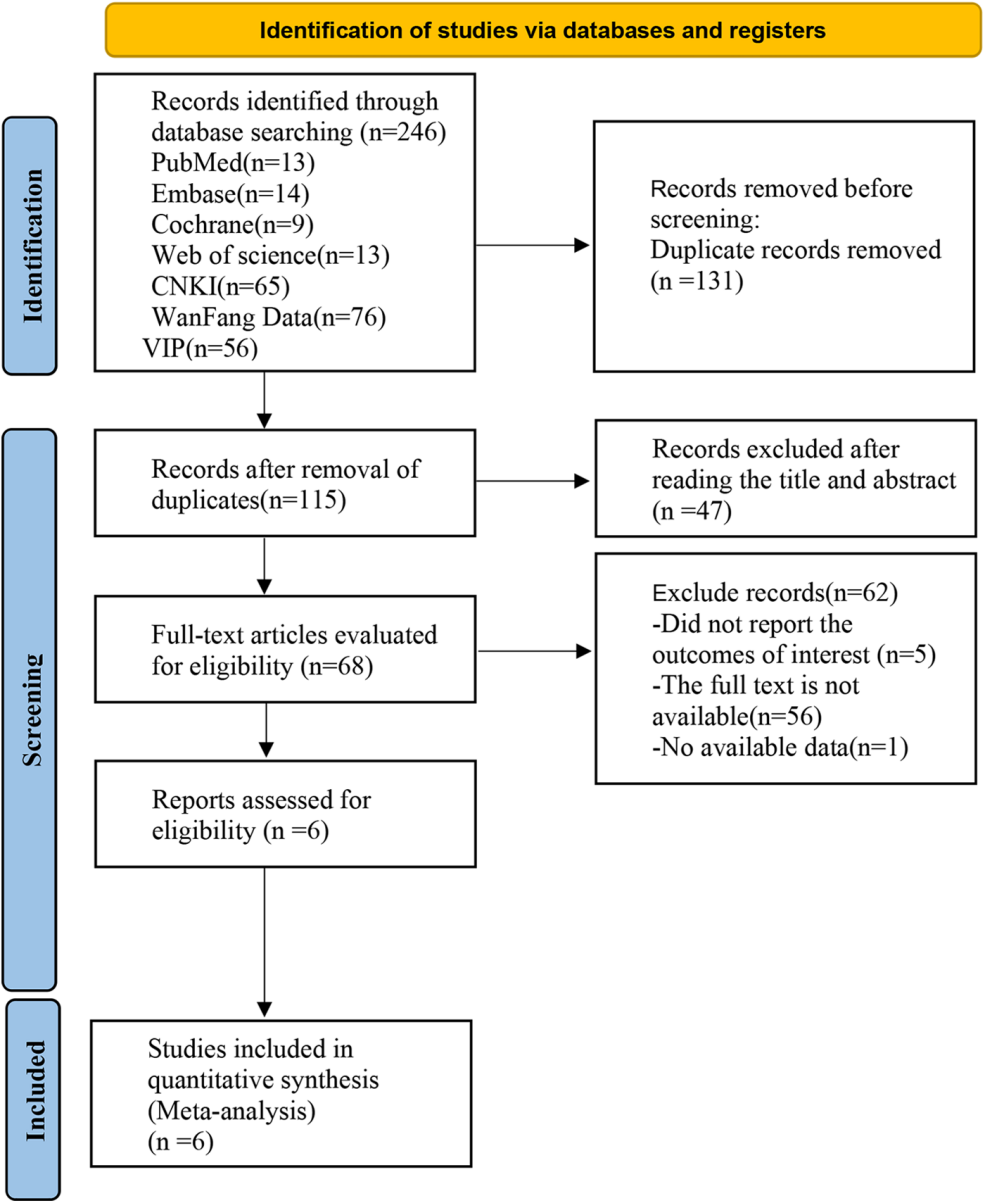
PRISMA flow diagram.

A total of 246 potentially relevant articles were identified through database searches: PubMed (*n* = 13), EMbase (*n* = 14), the Cochrane Library (*n* = 9), Web of Science (*n* = 13), CNKI (*n* = 65), WanFang Data (*n* = 76), and VIP (*n* = 56). After removing duplicate publications, 115 papers remained. Following the initial screening of titles and abstracts, 68 papers were deemed potentially eligible. However, a comprehensive review of the full texts revealed that 62 papers did not meet the predefined inclusion and exclusion criteria. Consequently, six RCTs (34–39), involving 767 patients, were ultimately incorporated in the analysis. The detailed screening process is illustrated in Figure 1.

### 3.2 Baseline characteristics and bias risk assessment results included in the study

**Figure 2:**
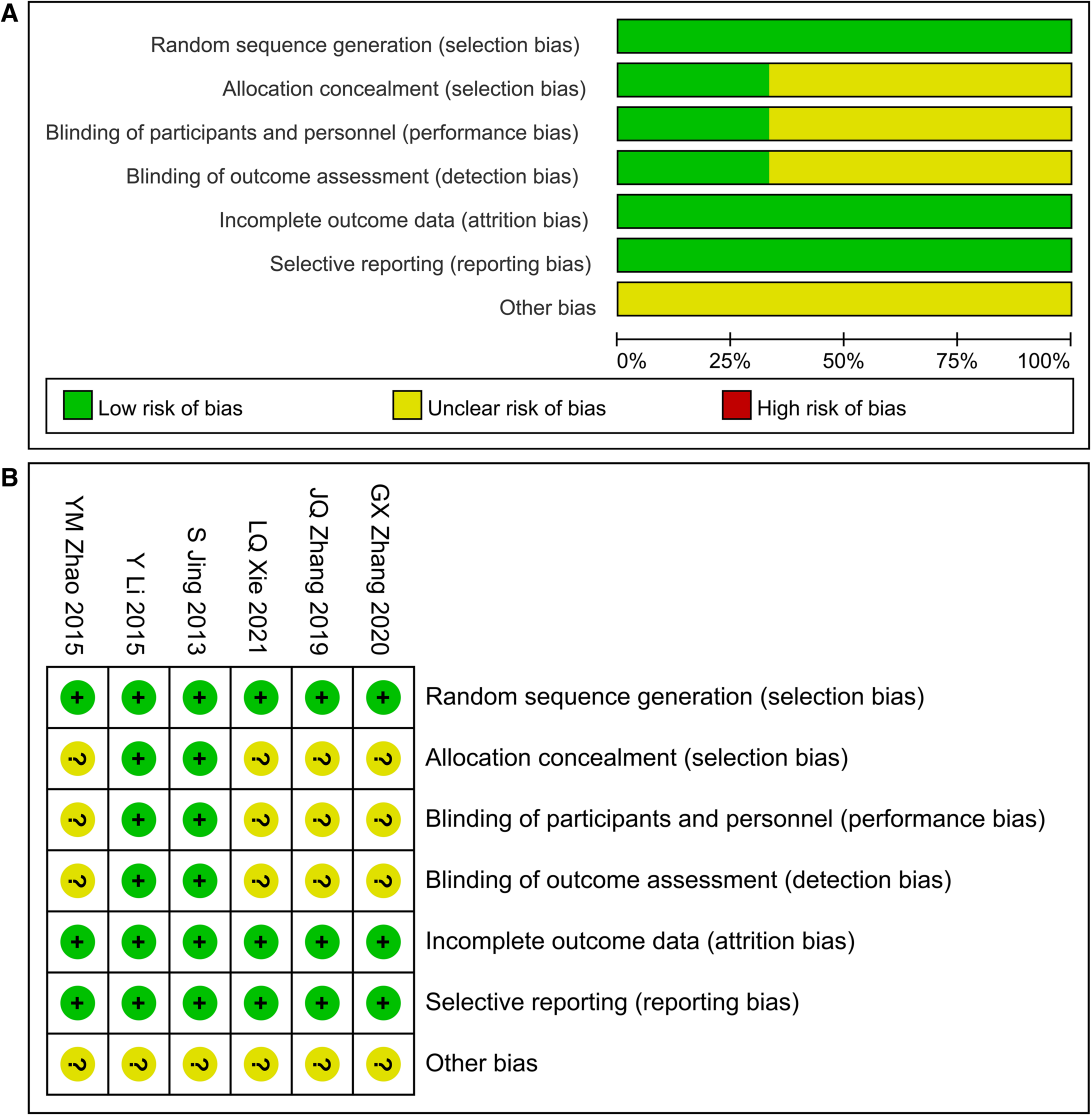
(**A**) Risk of bias graph; (**B**) risk of bias summary.

The analysis included six studies, three conducted in English and three in Chinese, all originating from China and involving a total of 767 participants, comprising 387 men and 380 women. All studies reported randomization, with two studies employing double-blind methods and one utilizing single-blind methods. Two studies compared Allisartan Isoproxil with placebo, while four studies compared it with conventional antihypertensive therapy alone. The dosage of Allisartan Isoproxil was consistent across five studies, administered at 240 mg once daily, with one study employing an 80 mg daily dose. Treatment duration ranged from 30 days to 6 months. Essential study characteristics are summarized in Table 1. Bias risk assessment was conducted using the Cochrane Collaboration's risk of bias tool, with outcomes depicted in Figures 2A,B.

**Table 1:** Basic characteristics of the included studies.

Study	Year	Sample size		Gender (M/F)	Conditions	Mean age		Intervention		Outcome	Follow up
		EG	CG			EG	CG	EG	CG		
S Jing	2013	72	70	78/64	None	54	54	Allisartan Isoproxil: 240 mg once daily	Losartan: 50 mg once daily	A1;A2; A6; A7	8 Weeks
Y Li	2015	137	138	117/158	None	54.2	55.4	Allisartan Isoproxil: 240 mg once daily	placebo	A1; A2; A6; A7	8 Weeks
YM Zhao	2015	45	45	45/45	CHD	-	-	Allisartan Isoproxil: 80 mg once daily	Irbesartan:150 mg once daily	A6; A7;	6 Weeks
JQ Zhang	2019	40	40	52/28	None	65.17	64.98	Allisartan Isoproxil: 240 mg once daily	nifedipine gastrointestinal therapeutic	A1; A2; A3; A4; A5	6 Months
									System:30 mg once daily		
GX Zhang	2020	34	34	35/33	No	42.7	41.2	Allisartan Isoproxil: 240 mg once daily	placebo	A1; A2; A3	30 days
LQ Xie	2021	59	53	67/45	CHD	53.01	52.89	Allisartan Isoproxil: 240 mg once daily	Irbesartan:150 mg once daily Or 300 mg	A4; A5; A6; A7	8 Weeks

A1: SBP, systolic blood pressure; A2: DBP, diastolic blood pressure; A3: baPWV, Brachial-Ankle Pulse wave velocity, A4: NO, nitric oxide; A5: ET, endothelin; A6: AE, adverse even; A7: The rate of effective blood pressure control; EG, Experimental Group; CG, Control Group.

### 3.3 Efficacy evaluation and outcome

#### 3.3.1 Systolic blood pressure

**Figure 3:**
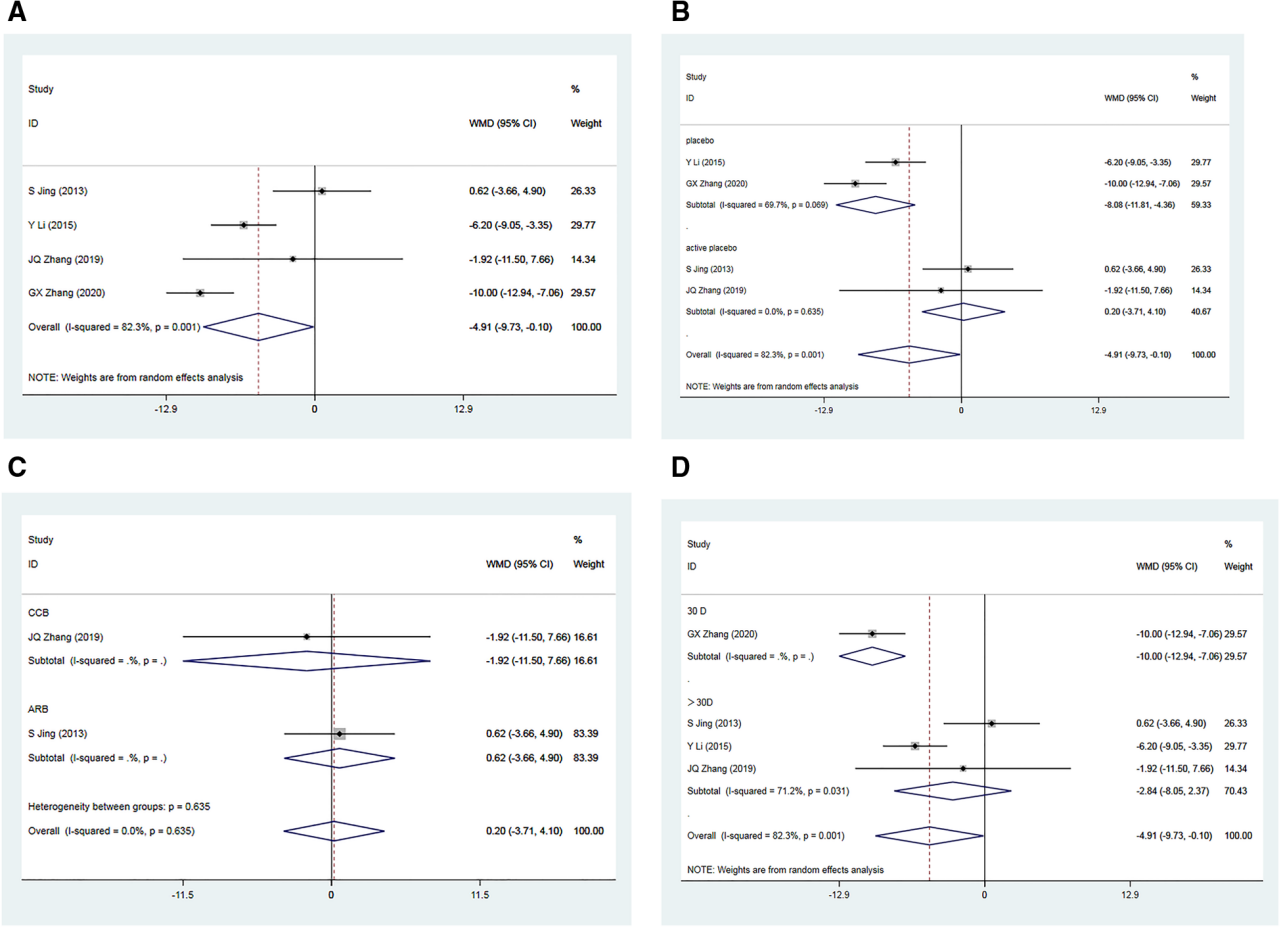
Forest plot for the meta-analysis of SBP. (**A**) Allisartan Isoproxil vs. non-Allisartan Isoproxil, (**B**) placebos vs. active placebos, (**C**) control group received CCB vs. control group received ARB, (**D**) 30 days follow-up vs. > exceeding 30 days follow-up.

In the analysis, four RCTs (34–37) involving a total of 535 patients reported post-treatment enhancements in SBP within the Allisartan Isoproxil group in comparison to the control group. However, considerable heterogeneity was detected among the studies (*I*² = 82.3%, *p* = 0.001). Meta-analysis findings indicated a more favorable reduction in SBP in the experimental group compared to the control group [WMD = −4.91, 95% CI (−9.73, −0.10), *p* = 0.045] (Figure 3A). Sequential sensitivity analysis showed that heterogeneity remained largely unaffected.

Subgroup analysis was carried out based on the presence of a placebo in the control group. Among the included RCTs, two of them employed a placebo in the control group (34, 36). Considerable heterogeneity was observed among these studies (*I*² = 69.7%, *p* = 0.069). Meta-analysis revealed a notably greater reduction in SBP within the experimental group compared to the control group [WMD = −8.08, 95% CI (−11.81, 4.10), *p* = 0.000]. However, in two RCTs where the control group received an active placebo (35, 37), heterogeneity was minimal (*I*^2 ^= 0.00%, *p* = 0.639). The meta-analysis showed a marginally superior reduction in SBP within the experimental group compared to the control group [WMD = 0.20, 95% CI (−3.71, 4.10), *p* = 0.921], which was not statistically significant. Variability in drugs used in the control group may have contributed to the observed heterogeneity (Figure 3B).

Subgroup analyses were performed based on the use of non-placebo drugs in the control group. Among the included RCTS, two of the control groups used non-placebo, CCB (nifedipine) and ARB (Losartan). Meta-analysis results showed a significant reduction in SBP in the CCB group compared to the control group (35), but the difference was not statistically significant [WMD = −1.92, 95% CI (−11.50, 7.66), *p* = 0.695]. In the ARB group (37), the reduction in SBP in the experimental group was slightly better than that in the control group, but the difference was not statistically significant [WMD = 0.62, 95% CI (−3.66, 4.90), *p* = 0.776]. Subgroup analysis suggested that compared to CCB antihypertensive drugs, Allisartan Isoproxil had a more significant effect on reducing SBP, but compared to ARB drugs, the effect was not evident, though not statistically significant (Figure 3C).

Additionally, subgroup analysis based on varying follow-up durations was conducted. One RCT (34) with a 30-day follow-up showed a significant advantage in SBP reduction within the experimental group compared to the control group [WMD = −10.00, 95% CI (−12.94, −7.06), *p* = 0.000]. Three RCTs (35–37) with treatment durations exceeding 30 days exhibited considerable heterogeneity (*I*^2 ^= 71.2%, *p *= 0.031). Meta-analysis outcomes indicated a somewhat more favorable reduction in SBP within the experimental group compared to the control group, but the differences were not statistically significant [WMD = −2.84, 95% CI (−8.05, 2.37), *p *= 0.286]. Subgroup analysis suggested that variations in intervention duration might contribute to heterogeneity, and minimal differences were observed when treatment duration exceeded 1 month (Figure 3D).

#### 3.3.2 Diastolic blood pressure

A total of four RCTs (34–37) comprising 535 patients, reported post-treatment improvements in DBP in the Allisartan Isoproxil group vs. the control group. However, significant heterogeneity was observed among the included studies (*I*^2 ^= 94.1%, *p* = 0.000). Meta-analysis results indicated a more pronounced reduction in DBP in the experimental group compared to the control group [WMD = −2.72, 95% CI (−7.20, 1.76), *p* = 0.234], although this difference was not statistically significant. Sequential sensitivity analysis demonstrated that excluding individual studies did not substantially affect heterogeneity.

**Figure 4:**
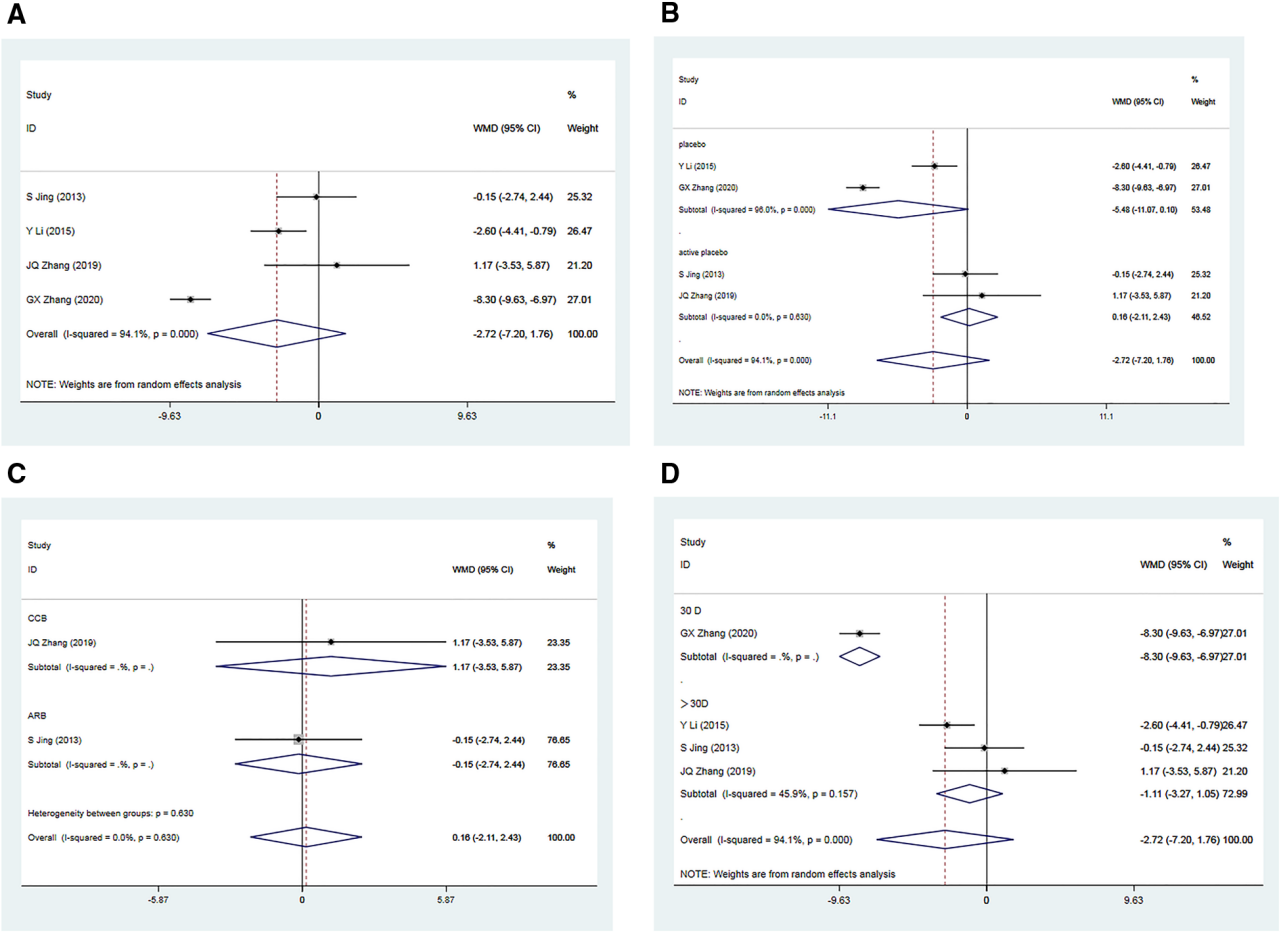
Forest plot for the meta-analysis of DBP. (**A**) Allisartan Isoproxil vs. non-Allisartan Isoproxil, (**B**) placebos vs. active placebos, (**C**) control group received CCB vs. control group received ARB, (**D**) 30 days follow-up vs. > exceeding 30 days follow-up.

A subgroup analysis was conducted based on whether the control group received a placebo. In two RCTs (34, 36) where the control group received a placebo, considerable heterogeneity was observed (*I*^2 ^= 96.0%, *p* = 0.000). Meta-analysis results showed a slightly more favorable reduction in DBP in the experimental group compared to the control group [WMD = −5.48, 95% CI (−11.07, 0.10), *p* = 0.054], but this difference was not statistically significant. Conversely, in two RCTs where participants in the control group received an active placebo, minimal heterogeneity was observed (*I*^2 ^= 0.00%, *p* = 0.630). The meta-analysis revealed no significant difference in DBP reduction between the experimental and control groups [WMD = 0.16, 95% CI (−2.11, 2.43), *p* = 0.891]. Subgroup analysis indicated that the drugs used in the control group were unlikely to be the primary cause of the observed heterogeneity (Figure 4B).

Subgroup analyses were performed based on the use of non-placebo drugs in the control group. When the control group received CCB antihypertensive drugs (Nifedipine) (35), the reduction in DBP in the experimental group was not significantly different from the control group [WMD = 1.17, 95% CI (−3.53, 5.87), *p* = 0.626]. Similarly, when the control group received ARB antihypertensive drugs (Losartan) (37), the reduction in DBP in the experimental group was not significantly different from the control group [WMD = −0.15, 95% CI (−2.74, 2.44), *p* = 0.910]. These findings suggest that Allisartan Isoproxil did not exhibit a significant advantage in reducing DBP compared to CCB and ARB antihypertensive drugs (Figure 4C).

Additionally, subgroup analysis based on varying follow-up durations was conducted. In the 30-day study (34), the experimental group exhibited a significantly superior effect in reducing DBP compared to the control group [WMD = −8.30, 95% CI (−6.93, −6.97), *p* = 0.000], with a highly significant difference. However, for studies with treatment durations exceeding 30 days (35–37), moderate heterogeneity was observed (*I*^2 ^= 45.9%, *p* = 0.157). In these cases, the reduction in DBP in the experimental group was only marginally superior to the control group [WMD = −1.11, 95% CI (−3.27,1.05), *p *= 0.313], with no statistically significant difference. Subgroup analysis suggests that variations in intervention duration could potentially explain the observed heterogeneity among studies, with minimal differences in outcomes observed when treatment duration exceeded one month (Figure 4D).

#### 3.3.3 Brachial-ankle pulse wave velocity

**Figure 5:**
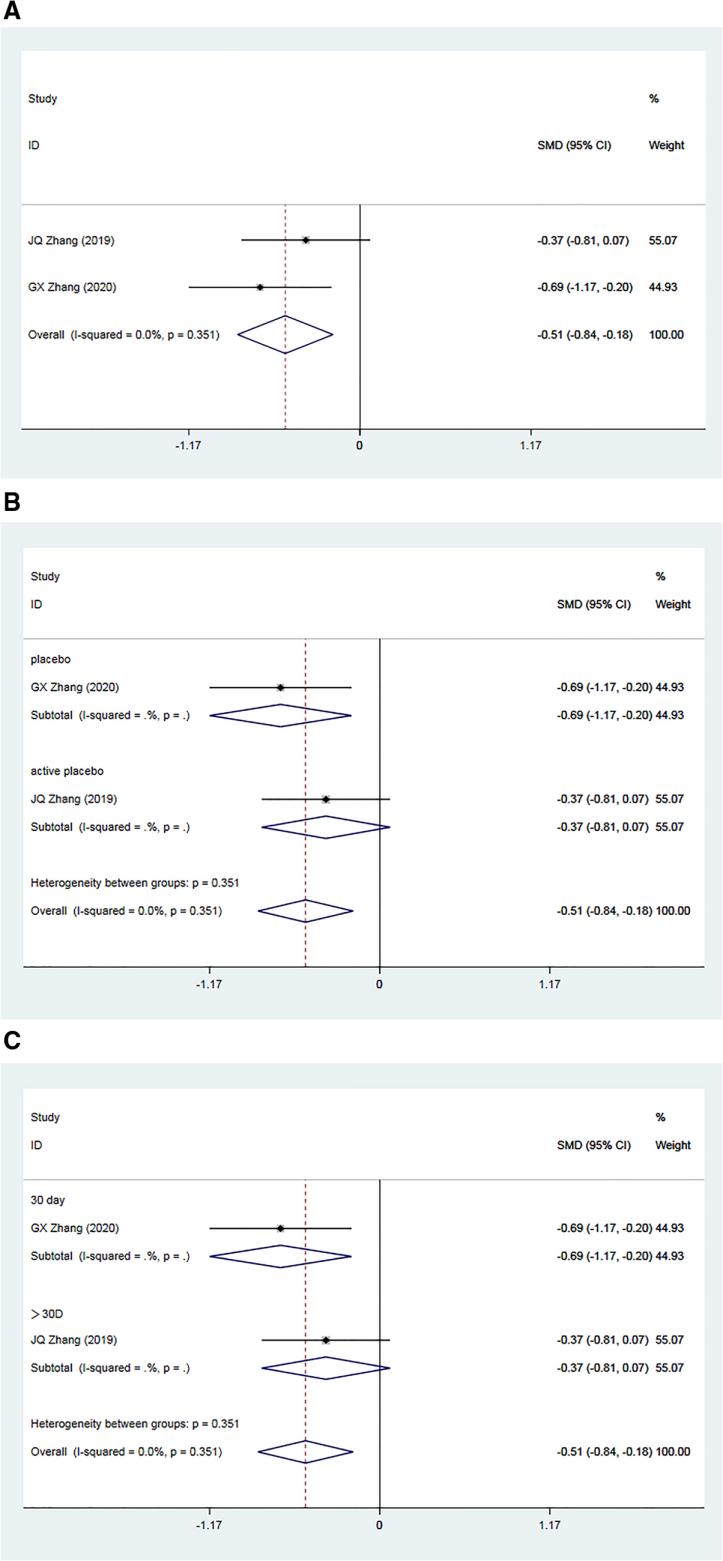
Forest plot for the meta-analysis of baPWV. (**A**) Allisartan Isoproxil vs. non-Allisartan Isoproxil, (**B**) placebos vs. active placebos, (**C**) 30 days follow-up vs. > exceeding 30 days follow-up.

A total of two RCTs (34, 35), including 148 patients, reported post-treatment improvements in baPWV in the Allisartan Isoproxil group vs. the control group. Analysis revealed minimal inter-study heterogeneity (*I^2^*^ ^= 0.0%, *p* = 0.351). Meta-analysis results demonstrated a significant reduction in baPWV favoring the Allisartan Isoproxil group over controls [SMD = −0.51, 95% CI (−0.84, −0.18), *p* = 0.002] (Figure 5A).

Subgroup analysis was carried out according to the type of placebo employed in the control group. In one study (34), the control group received a placebo, and meta-analysis results demonstrated a superior reduction in baPWV in the experimental group compared to the control group [SMD = −0.69, 95% CI (−1.17, 0.20), *p* = 0.006]. Conversely, in another study (35), the control group received an active placebo (nifedipine), showing a slightly better effect of baPWV reduction in the experimental group compared to controls [SMD = 0.37, 95% CI (−0.81, 0.07), *p* = 0.099]. This subgroup analysis suggests that heterogeneity may arise from variations in placebo interventions. Notably, the efficacy of Allisartan Isoproxil in reducing baPWV was more pronounced when the control group received a placebo (Figure 5B).

Further subgroup analysis based on follow-up durations was conducted. One study (34) had a follow-up duration of 30 days, demonstrating a significant baPWV reduction in the experimental group compared to controls [SMD = −0.69, 95% CI (−1.17, 0.20), *p* = 0.006]. Incorporating another study (35) with a treatment duration exceeding 30 days, meta-analysis outcomes showed a slight superiority of baPWV reduction in the experimental group compared to the observation group [SMD = 0.37, 95% CI (−0.81, 0.07), *p* = 0.099]. This subgroup analysis suggests that heterogeneity among studies could be attributed to differences in intervention duration, with minimal distinction in outcomes observed when treatment duration exceeded one month (Figure 5C).

#### 3.3.4 Nitric oxide

**Figure 6:**
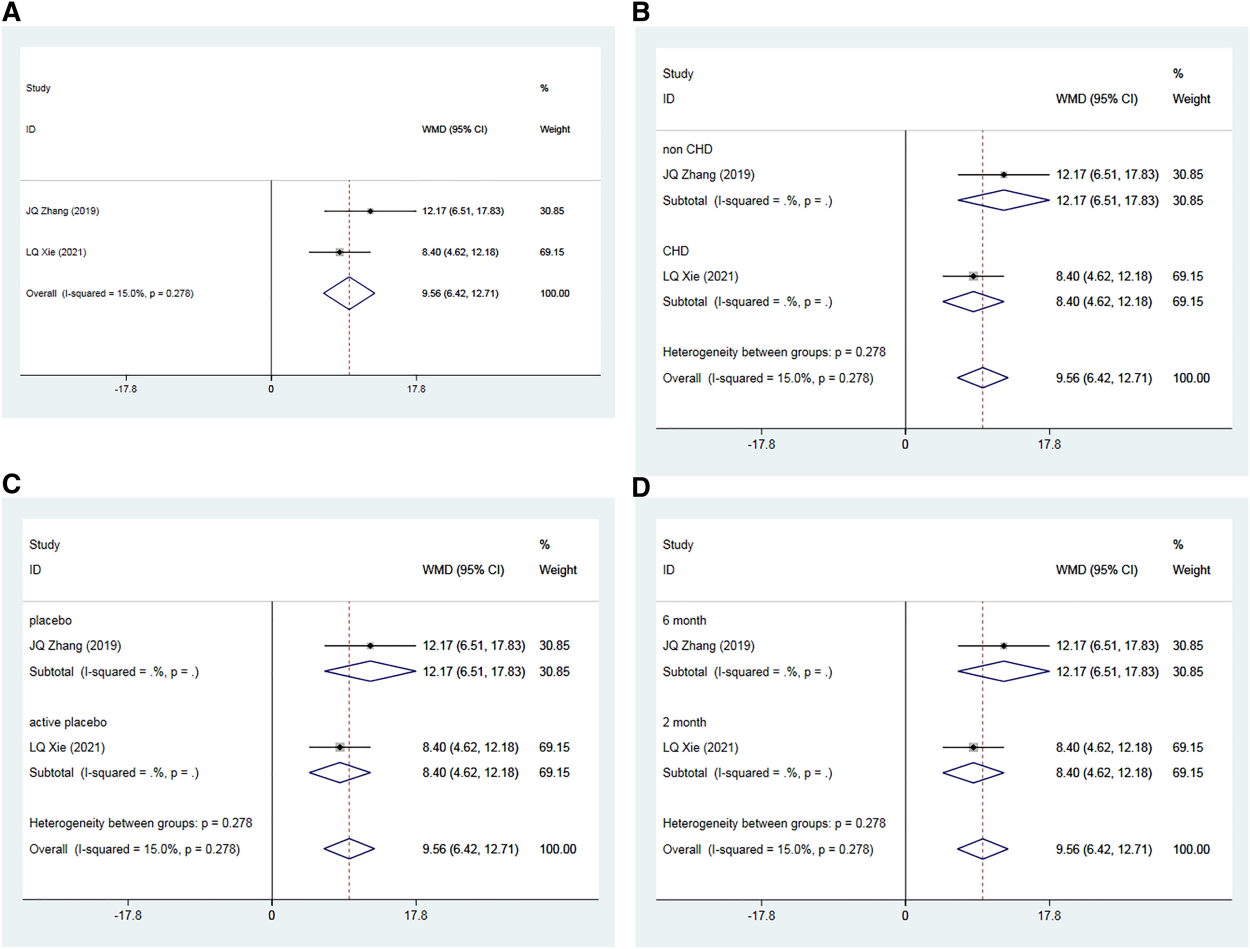
Forest plot for the meta-analysis of NO. (**A**) Allisartan Isoproxil vs. non-Allisartan Isoproxil, (**B**) hypertension with CHD vs. hypertension, (**C**) placebos vs. active placebos, (**D**) 6-month follow-up vs. 2-month follow-up.

A total of two RCTs (35, 39), including 192 patients, examined the effects of Allisartan Isoproxil on post-treatment improvements in NO levels compared to control groups. The heterogeneity test exhibited low inter-study heterogeneity (*I*^2 ^= 15.0%, *p* = 0.278). Meta-analysis results revealed a significant increase in NO levels favoring the experimental group over controls [WMD = 9.56, 95% CI (6.42, 12.71), *p* = 0.000] (Figure 6A).

Subgroup analysis was performed based on the presence or absence of CHD in the subjects. In one study (35) involving patients with simple hypertension, the meta-analysis showed a significantly better increase in NO levels in the experimental group compared to the control group [WMD = 12.17, 95% CI (6.51, 17.83), *p *= 0.000]. In the other study (39), which included patients with both hypertension and CHD, the experimental group also showed a significant improvement in NO levels compared to controls [WMD = 8.40, 95% CI (4.62, 12.18), *p* = 0.000] (Figure 6B).

Subgroup analysis considering the type of placebo used in the control group was also conducted. In the study where a placebo was used (35), the experimental group demonstrated a more pronounced effect on increasing NO levels compared to the control group [WMD = 12.17, 95% CI (6.51, 17.83), *p *= 0.000]. In the study where an active placebo (Irbesartan) was used (39), the experimental group again showed a significant increase in NO levels compared to the control group [WMD = 8.40, 95% CI (4.62, 12.18), *p* = 0.000] (Figure 6C).

Finally, subgroup analysis based on follow-up durations was conducted. In the 6-month study (35), the experimental group exhibited a more significant effect on increasing NO compared to controls [WMD = 12.17, 95% CI (−6.51, 17.83), *p* = 0.000]. In the 2-month study (39), the experimental group showed a slightly more pronounced effect on increasing NO compared to the observation group [WMD = 8.40, 95% CI (4.62, 12.18), *p* = 0.000] (Figure 6D).

#### 3.3.5 Endothelin

**Figure 7:**
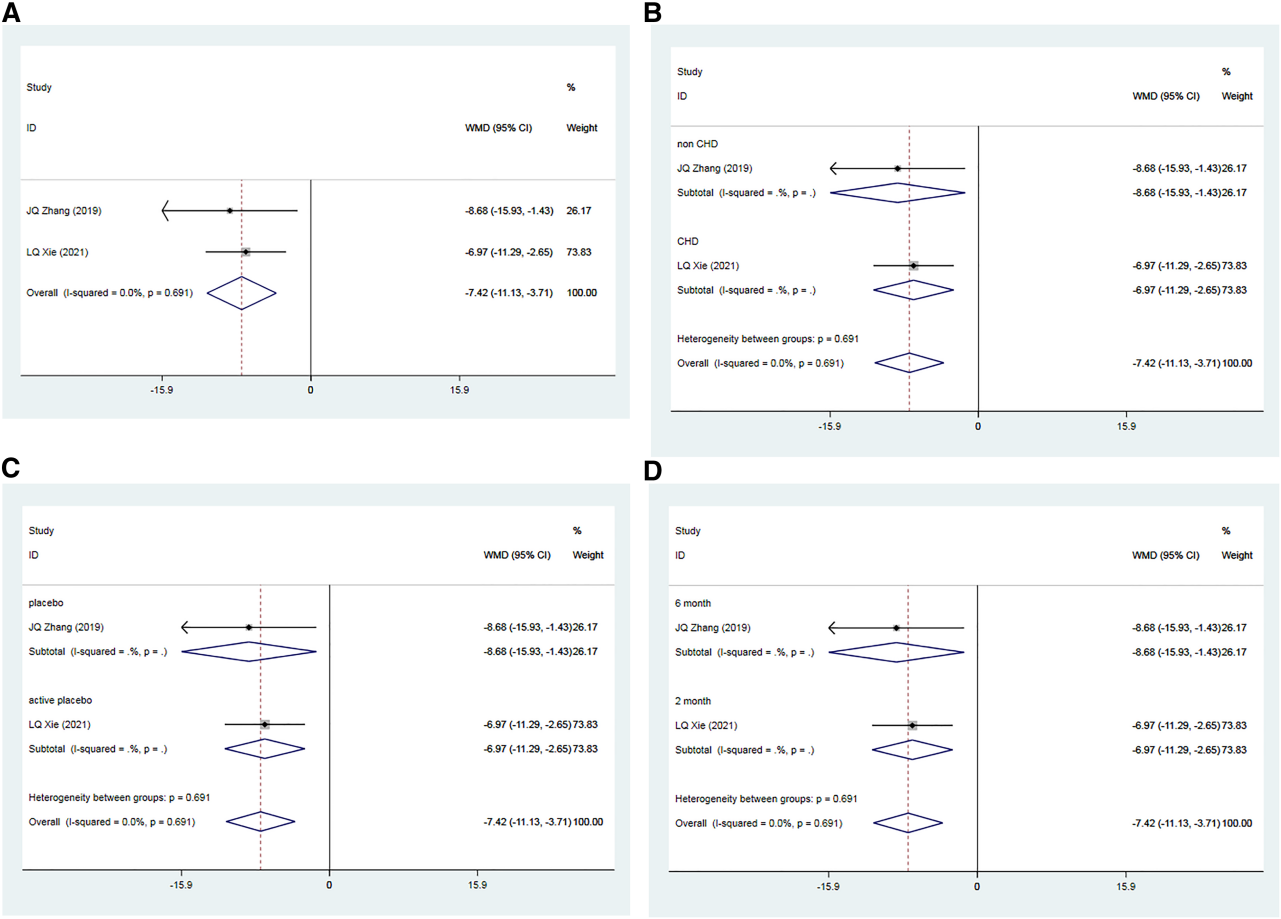
Forest plot for the meta-analysis of ET. (**A**) Allisartan Isoproxil vs. non-Allisartan Isoproxil, (**B**) hypertension with CHD vs. hypertension, (**C**) placebos vs. active placebos, (**D**) 6-month follow-up vs.2-month follow-up.

A total of two RCTs (35, 39), including 192 patients, investigated the impact of Allisartan Isoproxil on post-treatment improvements in ET levels compared to control groups. The heterogeneity test exhibited low inter-study heterogeneity (*I*^2 ^= 0.0%, *p* = 0.691). Meta-analysis findings indicated that the experimental group achieved a significantly greater reduction in ET levels compared to the control group [WMD = −7.42, 95% CI (−11.13, −3.71), *p* = 0.000] (Figure 7A).

Subgroup analysis was carried out according to whether the subjects had CHD or not. In one study (35) involving patients with uncomplicated hypertension, the experimental group demonstrated a significantly superior reduction in ET levels compared to controls [WMD = −8.68, 95% CI (−15.93, −1.43), *p* = 0.000]. In the other study (39), which included patients with both hypertension and CHD, the experimental group also demonstrated a significant reduction in ET levels compared to controls [WMD = −6.97, 95% CI (−11.29, −2.65), *p* = 0.000] (Figure 7B).

Subgroup analysis according to the type of placebo used in the control group was also conducted. In one study (35) employing a placebo, the experimental group exhibited a significantly lower reduction in ET levels compared to controls [WMD = 12.17, 95% CI (−6.51, 17.83), *p* = 0.000]. In the study where an active placebo (Irbesartan) was used (39), the experimental group again showed a significantly greater reduction in ET levels compared to the control group [WMD = 8.40, 95% CI (4.62, 12.18), *p* = 0.000] (Figure 7C).

Additionally, subgroup analysis based on follow-up duration was conducted. In the study with a 6-month follow-up (35), the experimental group demonstrated a significantly better reduction in ET levels compared to controls [WMD = 12.17, 95% CI (−6.51, 17.83), *p *= 0.000]. In the trial with a 2-month follow-up (39), the experimental group showed a slightly better reduction in ET levels compared to the observation group [WMD = −6.97, 95% CI (−11.29, −2.65), *p* = 0.000] (Figure 7D).

#### 3.3.6 The rate of effective blood pressure control

**Figure 8:**
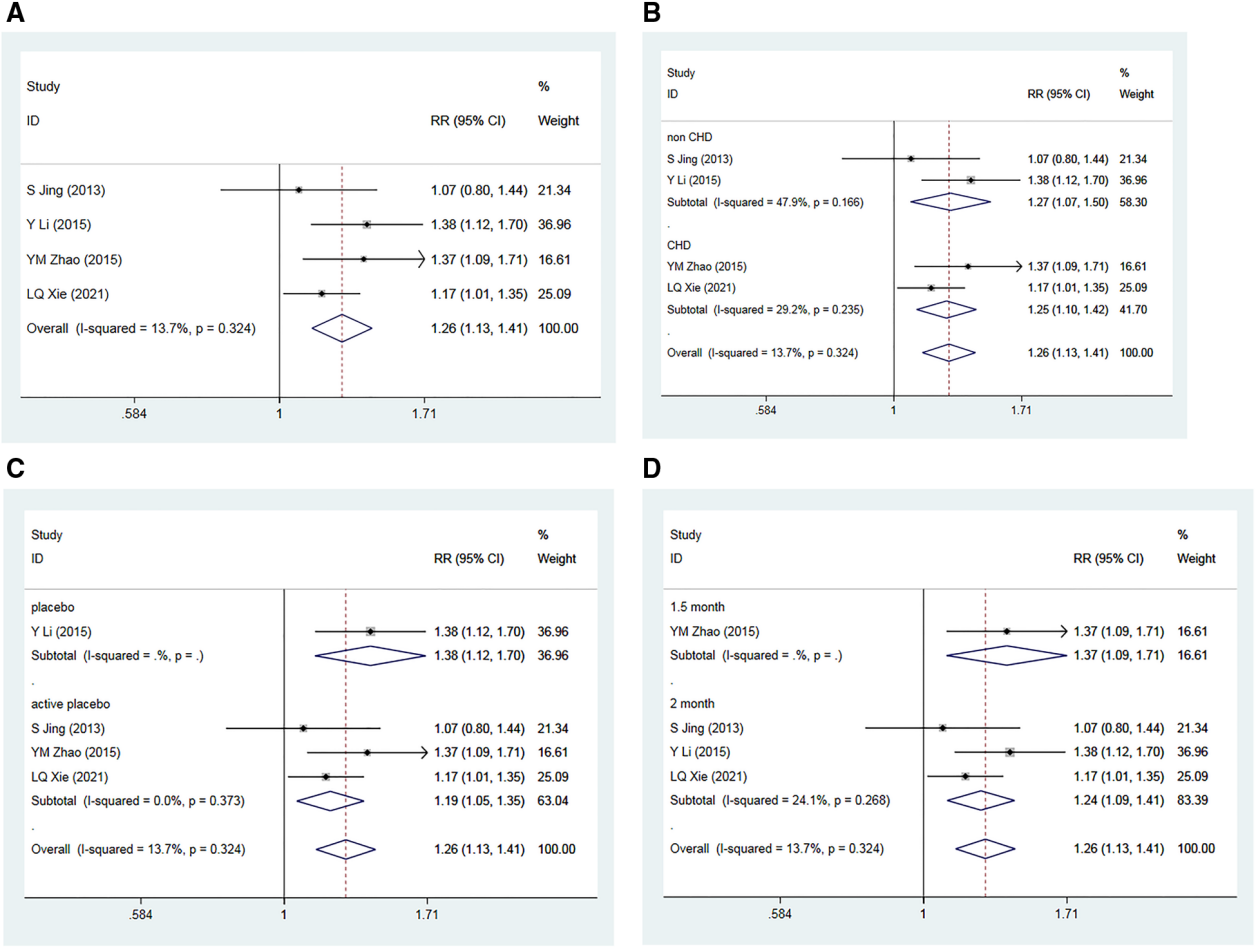
Forest plot for the meta-analysis of the rate of effective blood pressure controls. (**A**) Allisartan Isoproxil vs. non-Allisartan Isoproxil, (**B**) hypertension with CHD vs. hypertension, (**C**) placebos vs. active placebos, (**D**) 2-month follow-up vs.1.5-month follow-up.

Four RCTs (36–39), encompassing 619 patients, assessed the impact of Allisartan Isoproxil on the rate of successful blood pressure management compared to control groups. The heterogeneity test revealed low inter-study heterogeneity (*I*^2 ^= 13.7%, *p *= 0.324). Meta-analysis outcomes indicated that the experimental group achieved a significantly higher proportion of effective blood pressure control compared to the control group [RR = 1.26, 95% CI (1.13, 1.41), *p *= 0.000] (Figure 8A).

Subgroup analysis was conducted based on the presence of CHD in the subjects. For the two studies involving patients with simple hypertension (36, 37), moderate heterogeneity was observed (*I*^2 ^= 47.9%, *p *= 0.166). The meta-analysis findings implied that the proportion of effective blood pressure control in the experimental group was greater than in the control group [RR = 1.27, 95% CI (1.07, 1.50), *p* = 0.005]. For the two studies that involved hypertension and CHD patients (38, 39), low heterogeneity was observed (*I*^2 ^= 29.2%, *p* = 0.235), with the experimental group showing a slightly higher proportion of effective blood pressure control compared to controls [RR = 1.25, 95% CI (1.10, 1.42), *p* = 0.000] (Figure 8B).

Further subgroup analysis was performed based on the type of placebo used in the control group. In the one study using a placebo (36), the meta-analysis demonstrated that the experimental group had a greater proportion of effective blood pressure control compared to the control group [RR = 1.38, 95% CI (1.12, 1.70), *p* = 0.002]. In the three studies using active placebos (Irbesartan) (37–39), low heterogeneity was observed (*I*^2 ^= 0.0%, *p* = 0.373), with the meta-analysis showing a marginally higher proportion of effective blood pressure control in the experimental group compared to controls [RR = 1.19, 95% CI (1.05, 1.35), *p* = 0.007] (Figure 8C).

Subgroup analysis was also conducted according to the different follow-up time. Three studies with a 2-month follow-up (36, 37, 39) showed low heterogeneity (*I*^2 ^= 24.1%, *p* = 0.268). The meta-analysis results revealed that the proportion of effective blood pressure control in experimental group was greater compared to control group [RR = 1.24, 95% CI (1.09, 1.41), *p* = 0.001]. The study with a 1.5-month follow-up (38) demonstrated that the experimental group had a slightly higher proportion of successful blood pressure control compared to the control group [RR = 1.37, 95% CI (1.09, 1.71), *p *= 0.007] (Figure 8D).

#### 3.3.7 Adverse event

**Figure 9:**
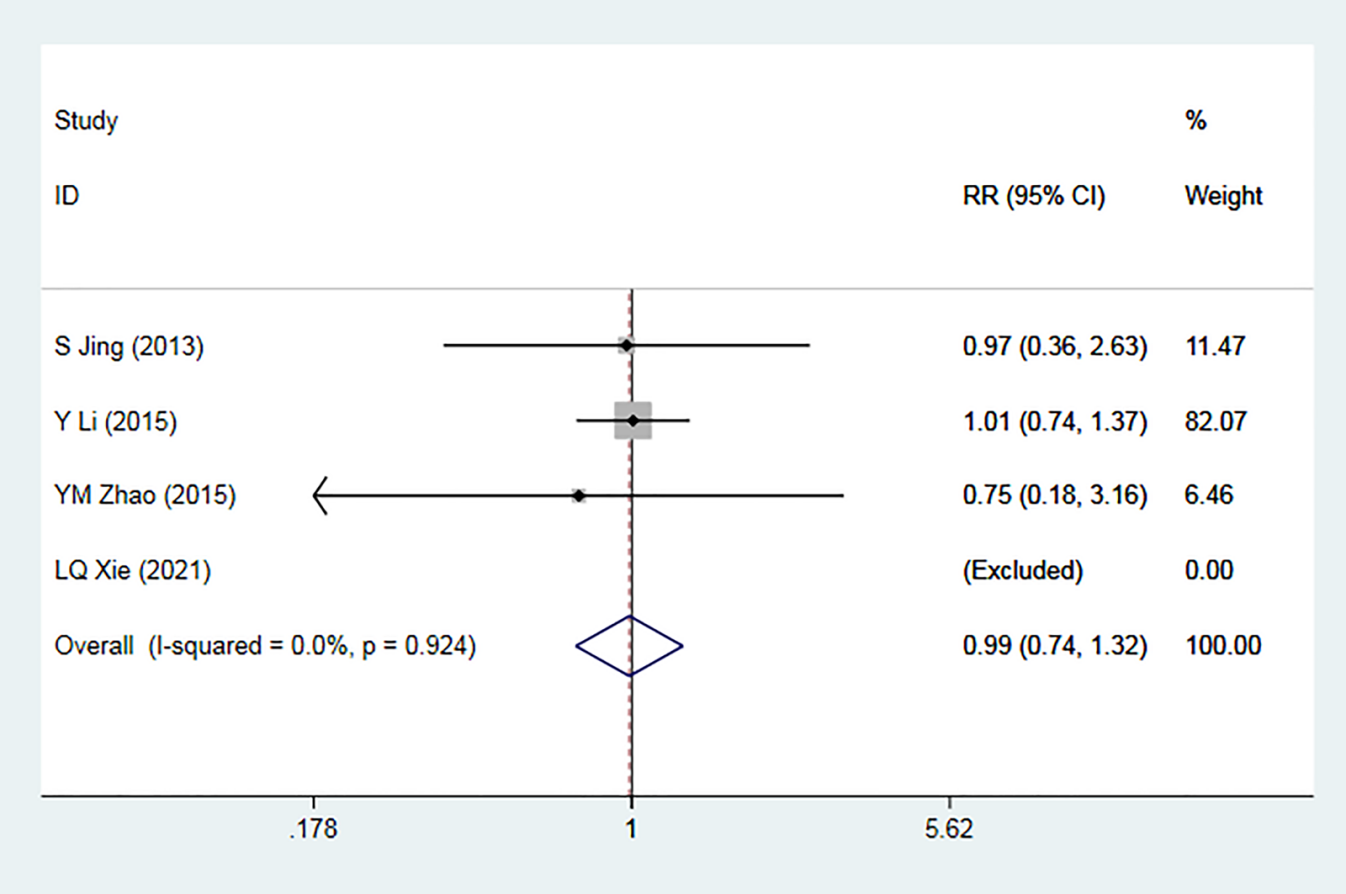
Forest plot for the meta-analysis of AE.

A comprehensive analysis was conducted, incorporating data from four RCTs (36–39) involving 619 patients. Among these studies, one reported no significant AE in either group, while three studies reported occurrences of AE. Importantly, none of the reported adverse events were fatal. Heterogeneity testing revealed low inter-study heterogeneity (*I*^2 ^= 13.7%, *p *= 0.924). The meta-analysis outcomes indicated no statistically significant difference in the incidence of adverse events between the experimental and control groups [RR = 0.99, 95% CI (0.74, 1.32), *p* = 0.928] (Figure 9).

Subgroup analysis was conducted, considering factors such as the presence of coronary heart disease, the type of placebo used, and follow-up duration in the control group. Across these subgroups, the meta-analysis consistently showed no statistically significant difference in the occurrence of adverse reactions between the experimental and control groups.

### 3.4 Sensitivity analysis

A sensitivity analysis was performed by systematically excluding individual studies one at a time. This analysis revealed no significant changes in the results, indicating the stability of the meta-analysis findings.

### 3.5 Publication bias

**Figure 10:**
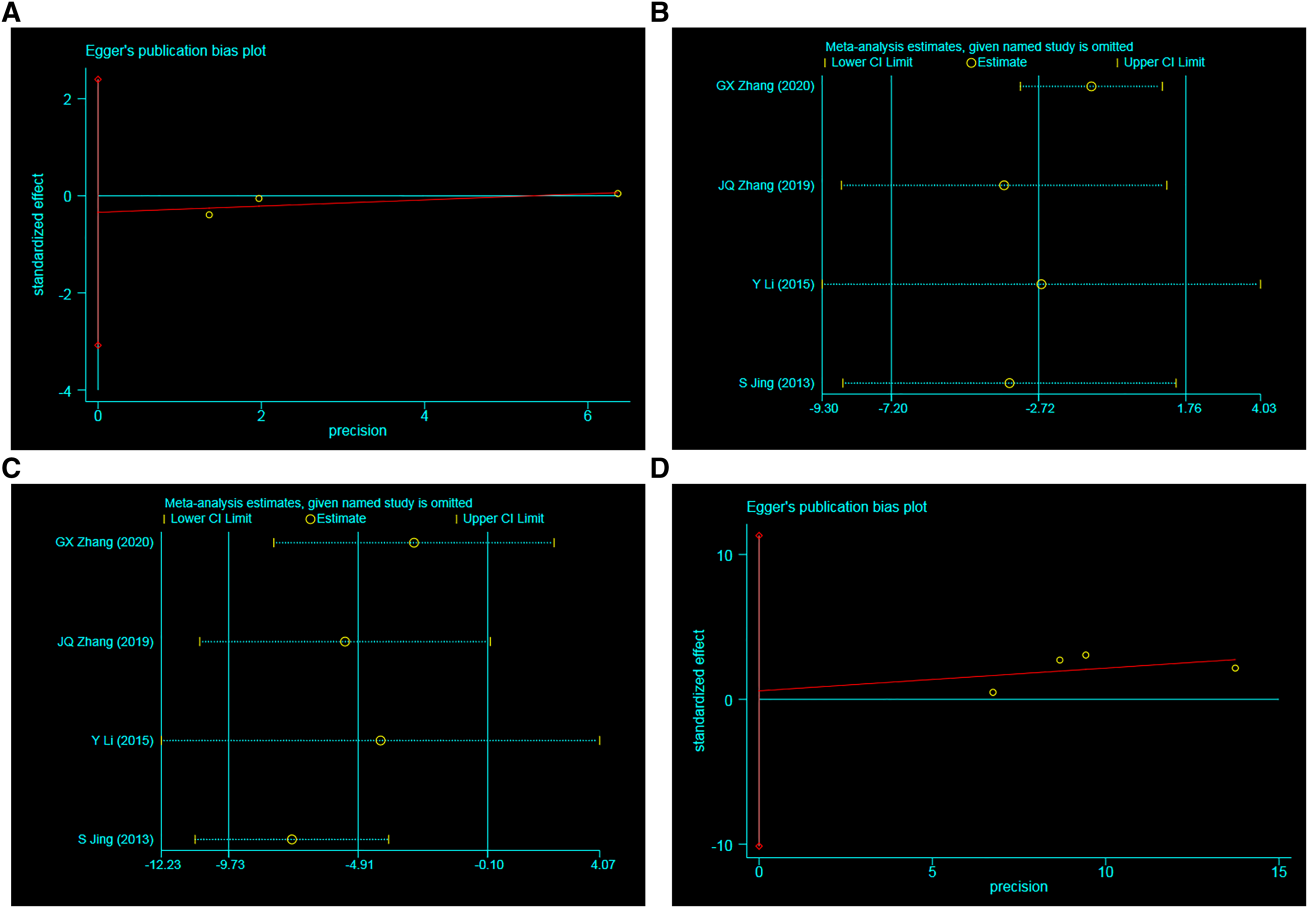
Egger's regression chart. (**A**) SBP, (**B**) DBP, (**C**) the total effective rate, (**D**) AE.

To examine publication bias, Egger's regression chart was generated for SBP, DBP, the rate of effective blood pressure control, and the incidence of adverse events. The Egger's test results indicated no significant publication bias for SBP (*t* = 0.90, *p* = 0.461), DBP (*t* = 1.95, *p* = 0.191), the total effective rate (*t* = 0.24, *p* = 0.836), and the incidence of adverse reactions (*t* = −1.58, *p* = 0.360). These findings suggest that publication bias did not significantly influence the results (Figure 10).

### 3.6 GRADE evidence quality grading results

**Figure 11:**
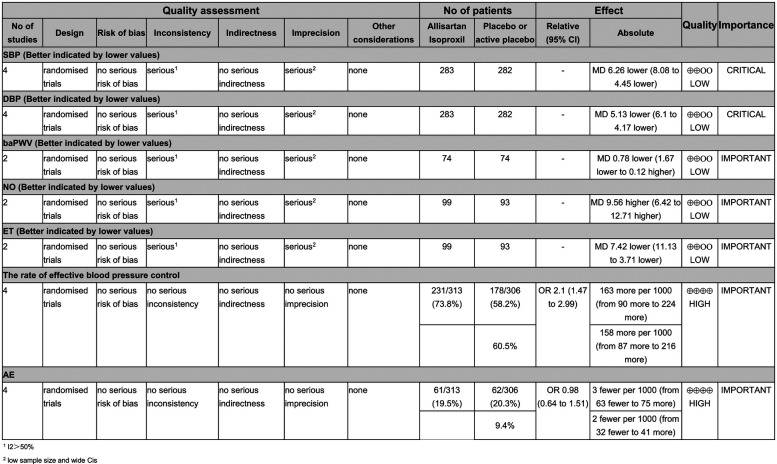
Summary table of GRADE evidence quality classification.

The quality of evidence for the outcome indicators related to the treatment of hypertension with Allisartan Isoproxil was assessed using the GRADE system. The evidence quality for “SBP,” “DBP,” “baPWV,” “NO,” and “ET” was rated as “low.” In contrast, the quality of evidence for “AE” and “the rate of effective blood pressure control” was rated as “high” (Figure 11).

## 4 Discussion

Our meta-analysis aimed to evaluate both the efficacy and safety of Allisartan Isoproxil in managing patients with essential hypertension, pooling data from six RCTs involving a total of 767 participants. The results demonstrate significant reductions in SBP, decreased baPWV, elevated NO levels, and decreased ET levels in patients treated with Allisartan Isoproxil compared to those receiving placebo or active placebo. However, the impact on reducing DBP was less pronounced, and did not reach statistical significance. Additionally, the administration of Allisartan Isoproxil led to an increased rate of effective blood pressure management. Importantly, the safety analysis revealed no statistically significant differences in the incidence of adverse events between the Allisartan Isoproxil group and the control group, with no fatal adverse events reported. Sensitivity analysis further confirmed the robustness of these findings.

All trials included in this meta-analysis reported the efficacy of Allisartan Isoproxil on blood pressure outcomes. Four studies detailed SBP and DBP outcomes, while two studies provided data on the effective blood pressure control rate. Our findings corroborate previous research indicating the efficacy of Allisartan Isoproxil in reducing SBP. Subgroup analysis showed a significant reduction in SBP compared to placebo, although no statistically significant differences were observed when comparing Allisartan Isoproxil to active placebos, including CCB drug (Nifedipine) and ARB drug (Losartan). Similarly, no statistically significant difference was found in DBP reduction between the Allisartan Isoproxil and control groups. This suggests that Allisartan Isoproxil, as a single agent, does not significantly outperform CCBs and ARBs in reducing SBP or DBP.

Notably, our analysis revealed a higher effective control rate for blood pressure in the Allisartan Isoproxil group compared to controls, with statistically significant results across all subgroups. All included studies used office blood pressure. However, the latest guidelines recommend assessing blood pressure control using non-office settings, such as home or dynamic blood pressure monitoring (40, 41). Non-office blood pressure monitoring may yield different results, such as lower blood pressure readings, especially after eliminating the effect of white coat hypertension, and can provide more stable blood pressure changes. Therefore, the antihypertensive effect of Allisartan Isoproxil may be better assessed using non-office blood pressure monitoring.

In a research by Wu et al, the result showed that Allisartan Isoproxil has the same antihypertensive effect as other marketed sartan drugs (42). Furthermore, evidence from Phase II and III multi-center, randomized, double-blind, placebo-controlled clinical trials suggests that Allisartan Isoproxil is particularly suitable for managing mild to moderate essential hypertension (36). Guidelines for hypertension management commonly recommend combination therapy involving a renin-angiotensin system inhibitor with a calcium channel blocker or combined diuretic (43). A multicenter, prospective, open phase IV clinical trial conducted by Wang et al. (27) investigated the antihypertensive efficacy of Allisartan Isoproxil combined with indapamide or amlodipine in patients who did not meet monotherapy standards. From baseline to 12 weeks, SBP decreased by 19.1 ± 11.7 mmHg and DBP decreased by 10.8 ± 8.7 mmHg. This indicates that Allisartan Isoproxil monotherapy can be an effective treatment for mild to moderate essential hypertension. For patients who do not respond well to single-drug therapy, combining Allisartan Isoproxil with other antihypertensive medications may be a viable therapeutic option. This underscores the potential of Allisartan Isoproxil as part of combination therapy in effectively managing hypertension, particularly in cases where monotherapy is insufficient.

The assessment of antihypertensive drugs should extend beyond conventional measures like SBP and DBP to encompass a broader array of vascular health markers. Hypertension often precipitates structural and functional changes in blood vessels, including endothelial dysfunction, vascular inflammation, and increased arterial stiffness (44). These vascular changes are closely linked with hypertension and may contribute to organ damage (45). Markers such as NO and ET are critical indicators of vascular endothelial dysfunction in hypertensive individuals. They reflect diminished basal and agonist-dependent NO release and heightened endogenous ET-induced vasoconstriction (46). Additionally, baPWV serves as an indicator of arterial stiffness in both central and peripheral arteries (47), which is clinically relevant due to its association with an increased risk of coronary heart disease (48).

In an animal study conducted by Arash et al, eight clinically available ARBs were tested. The study demonstrated that ARBs could enhance the activity of myocardial antioxidant enzymes, up-regulate nitric oxide synthase, and promote the release of NO. The authors suggested that ARBs may directly activate endothelial function through pathways independent of blood pressure lowering mechanisms (49). Another experiment on rats with renal vascular hypertension showed that Allisartan Isoproxil could stably reduce blood pressure, inhibit the angiotensin-aldosterone system, block oxidative stress pathways, and significantly reduce cerebrovascular injury incidence and severity. This study highlighted Allisartan Isoproxil's potential in preventing hypertensive stroke (50). At the same time, Allisartan Isoproxil can antagonize RAAS activity in local tissues and circulating tissues, inhibit the production of vascular inflammatory factors, reverse vascular wall function and structural abnormalities, and improve endothelial function (51). Animal experiments have also demonstrated its efficacy in alleviating diabetic cardiomyopathy by reducing oxidative stress and inflammation induced by diabetes (52). Our meta-analysis findings indicate that treatment with Allisartan Isoproxil significantly increases NO levels, decreases ET levels, and improves baPWV levels in hypertensive patients across various subgroups. Although the reduction in baPWV compared to active placebo did not reach statistical significance, the overall improvement in vascular health markers suggests that Allisartan Isoproxil may protect target organs and reduce the incidence of cardiovascular diseases by improving vascular endothelial function.

AE associated with Allisartan Isoproxil were reported in four out of the six studies included in our analysis. The most common AEs included hyperlipidemia, dizziness, headache, nausea, and abnormal liver function, with no fatal AEs observed. Importantly, our analysis found no significant differences in the occurrence of AEs between the Allisartan Isoproxil group and the control group across all subgroups. The lack of statistical significance in AE occurrence may be attributed to the relatively small sample sizes in the studies. It is noteworthy that only 14% of oral losartan doses are converted to EXP3174 by CYP2C9 and CYP3A4, and other metabolites may potentially lead to side effects and toxicity (53). However, Allisartan Isoproxil is meticulously formulated as an esterified prodrug, and its metabolism does not involve any cytochrome P450 subfamilies (54). It follows a simple metabolic pathway, resulting in the formation of a single metabolite, EXP3174. The metabolites would be excreted through bile and stool, but rarely through urine, reducing the burden of liver and kidney (28). In addition, EXP3174 is mainly distributed in tissues such as digestive tract and lung, and its content is very low in brain tissue, indicating limited penetration of the blood-brain barrier (55). Safety studies conducted on healthy Chinese subjects have confirmed the favorable safety profile of Allisartan Isoproxil, even at high doses (240 mg single dose and 480 mg multiple doses), with no serious AEs reported (56, 57). Therefore, Allisartan Isoproxil exhibits fewer adverse drug reactions and can be considered safe for use alone or in combination therapy.

Allisartan Isoproxil has been approved and put into use in many countries and regions, but there are still large regional differences in its use, particularly in China. Patent status, economic cost, and medical policies across different regions may all affect the use of Allisartan Isoproxil in clinical practice worldwide. The half-life of Allisartan Isoproxil is about 10 h, and the antihypertensive effect can last for 24 h. Because Allisartan Isoproxil is a precursor drug, its antihypertensive effect is smooth and slow compared with other ARB drugs. It takes 2 to 4 weeks to achieve the best antihypertensive effect. In addition, Allisartan Isoproxil can reduce uric acid. A previous study has shown that after continuous administration of Allisartan Isoproxil for three months, uric acid level is reduced by 24.2 mg/g, which has significant clinical significance (28). The mechanism of uric acid reduction by Allisartan Isoproxil is still unclear, but it may involve inhibition of uric acid transporters in renal tubules, improvement of insulin resistance, and reduction of uric acid production.

In our meta-analysis, which pooled data from six RCTs, we utilized the GRADE system to assess the quality of evidence for each outcome measure. The quality of evidence of primary outcomes such as “SBP” and “DBP” was categorized as “low” due to the high heterogeneity between studies. Furthermore, the relatively small sample size of the included studies and the wide CIs of the estimates may lead to inconsistent and inaccurate research results. This rating suggests that while there may be evidence supporting the efficacy of Allisartan Isoproxil in reducing blood pressure, further research is needed to strengthen the evidence base. For the same reason, secondary outcomes, including “baPWV”, “NO”, and “ET”, were also assessed as having “low” level evidence. While similar studies support the notion that Allisartan Isoproxil could positively impact the structure and function of the vascular endothelium, caution is warranted in drawing definitive conclusions based solely on the available data (58, 59).

In terms of safety assessment, the reports of AEs in various studies showed high consistency, resulting in the level of evidence for safety outcomes being rated as “high.” Allisartan Isoproxil demonstrated good safety characteristics. In future work, more large-scale, multi-center studies are needed to further improve the quality of evidence and provide a more solid foundation for clinical decision-making.

## 5 Limitations

Before applying the findings of this study to clinical practice, it's essential to consider several limitations. Firstly, the small number of included studies and their limited sample sizes may compromise the robustness of the results. Secondly, the treatment periods in the included studies were relatively short compared to the lifelong nature of hypertension management. Longer observation periods are necessary to confirm efficacy. Thirdly, there was heterogeneity among the included RCTs regarding the populations with hypertension and the interventions used, which introduces potential confounding factors. Although subgroup and sensitivity analyses were conducted to address this issue, it remains a concern. Fourthly, the included studies focused solely on office blood pressure measurements. This approach does not eliminate the effect of white coat hypertension and does not provide additional blood pressure variability data. Multi-dimensional blood pressure assessments, such as home or ambulatory monitoring, are needed to enhance the generalizability and applicability of the results. Lastly, all included studies were conducted in China, which may affect the international applicability and universality of the results. Differences in cultural contexts, medical practices, and study designs could influence the outcomes. These limitations underscore the need for cautious interpretation of the results and highlight the necessity for further research to address these constraints and enhance the applicability of the findings to broader clinical contexts.

## 6 Conclusion

This systematic review and meta-analysis, comprising six RCTs, demonstrate that Allisartan Isoproxil effectively reduces SBP and protects vascular endothelial function. Allisartan Isoproxil emerges as an effective and well-tolerated treatment for hypertension. However, these conclusions should be interpreted with caution due to the limited quantity and quality of the included studies. Additional cross-border, multilingual collaborations are needed to improve the generalizability of the research findings and provide deeper insights into the long-term efficacy and safety profile of Allisartan Isoproxil. Such investigations are essential for establishing its potential as a valuable therapeutic option for hypertension management.

## Data Availability

The original contributions presented in the study are included in the article/**Supplementary Material**, further inquiries can be directed to the corresponding author.
