# Supplementary material for: Efficacy and safety evaluation of Allisartan Isoproxil in patients with hypertension: a meta-analysis
Source: Front Cardiovasc Med. 2024 Jun 6;11:1355014. doi: 10.3389/fcvm.2024.1355014 (PMC11187348; doi:10.3389/fcvm.2024.1355014)
Supplement: Supplementary file 1 [file Table1.docx]

**Table S1 Detailed search strategy of target databases.**

**Search strategy of PubMed**

| No. | Query | Results |
| --- | --- | --- |
| #4 | (#1 OR #2) AND (#3) | 13 |
| #3 | (((allisartan isoproxil) OR (ALS-3 compound))) OR (allisartan isoproxil[Supplementary Concept]) | 16 |
| #2 | "Hypertension"[Title/Abstract] OR "Blood Pressure, High"[Title/Abstract] OR "Blood Pressures, High"[Title/Abstract] OR "High Blood Pressure"[Title/Abstract] OR "High Blood Pressures"[Title/Abstract] | 486,787 |
| #1 | "hypertension"[MeSH Terms] | 323,216 |

**Search strategy of Embase**

| NO. | Query | Results |
| --- | --- | --- |
| #5 | #3 AND #4 | 14 |
| #4 | #1 OR #2 | 1282911 |
| #3 | 'allisartan isoproxil':ti,ab,kw OR 'als-3 compound':ti,ab,kw | 18 |
| #2 | 'hypertension':ti,ab,kw OR 'blood pressure, high':ti,ab,kw OR 'blood pressures, high':ti,ab,kw OR 'high blood pressure':ti,ab,kw OR 'high blood pressures':ti,ab,kw | 782955 |
| #1 | 'hypertension'/exp | 1051397 |

**Search strategy of Cochrane library**

| NO. | Query | Results |
| --- | --- | --- |
| #1 | MeSH descriptor: [Hypertension] explode all trees | 26619 |
| #2 | (Hypertension):ti,ab,kw OR (Blood Pressure, High):ti,ab,kw OR (Blood Pressures, High):ti,ab,kw OR (High Blood Pressure):ti,ab,kw OR (High Blood Pressures):ti,ab,kw | 95428 |
| #3 | #1 OR #2 | 95429 |
| #4 | (allisartan isoproxil):ti,ab,kw OR (ALS-3 compound):ti,ab,kw | 13 |
| #5 | #3 AND #4 | 9 |

**Search strategy of Web of science**

| NO. | Query | Results |
| --- | --- | --- |
| #1 | TS=(Hypertension) OR TS=(Blood Pressure, High) OR TS=(Blood Pressures, High) OR TS=(High Blood Pressure) OR TS=(High Blood Pressures) | 648139 |
| #2 | TS=(allisartan isoproxil) OR TS=(ALS-3 compound) | 23 |
| #3 | #2 AND #1 | 13 |
